# The Effects of Polymerization on the Performance of Viologen-Based Electrochromic Devices

**DOI:** 10.3390/gels10110694

**Published:** 2024-10-25

**Authors:** Antonello Nucera, Carmen Rizzuto, Mario Michele Pipita, Irene Barba Castagnaro, Roberto Termine, Riccardo C. Barberi, Marco Castriota

**Affiliations:** 1 Department of Physics, University of Calabria, Via Ponte Bucci, Cubo 33B, 87036 Rende, CS, Italy; 2 CNR-Nanotec c/o, Department of Physics, University of Calabria, Via Ponte Bucci, Cubo 33B, 87036 Rende, CS, Italy

**Keywords:** ethyl viologen diperchlorate, ferrocene, bisphenol A glycerolate (1 glycerol/phenol) diacrylate, electrochromic device, polymeric gel electrolyte, Raman spectroscopy, cyclic voltammetry, UV-vis-Nir spectroscopy

## Abstract

In this work, electrochromic devices were prepared using the redox couple ethyl viologen diperchlorate and 1,1′-diethyl ferrocene in propylene carbonate as an aprotic solvent to facilitate ions separation and diffusion inside the devices. Electrochromic devices were made using electrochromic gel mixtures at the concentrations of 55%, 60% and 65% with respect to the bisphenol A polymer. In particular, two sets of gels were made: one set contained the bisphenol A not-polymerized while and the second one contained the polymerized polymer. Different techniques, such as cyclic voltammetry, UV-vis-NIR, and Raman spectroscopy, were used to study such systems to understand the differences in terms of performances between the different sets of electrochromic devices. Cyclic voltammetry confirmed that the oxidation process of the 1,1′-diethyl ferrocene and the reduction of the ethyl viologen diperchlorate occurred at about 0.4 V. Interesting variations in the transmittances were found between the two groups of samples. The best values of CE were provided by the electrochromic devices based on the polymerized electrochromic gel mixture at a concentration of 60% (EM_60_). The EM_60_ device result was CE = 92.82 C/cm^2^ in the visible region and CE = 80.38 C/cm^2^ in the near–infrared region, confirming that these devices can be used for energy-saving applications. A structural characterization of the materials used in the two sets of electrochromic devices was made using Raman spectroscopy, and the analysis supports the electrochemical models used to explain the processes involved during operation of the electrochromic systems.

## 1. Introduction

Electrochromism is the phenomenon linked to a reversible change in the color of a device following a redox reaction, which occurs after the application of an external voltage to the device. Interest in electrochromic materials is, recently, increased significantly because of their possible applications in smart windows, displays [1] and in building energy-saving applications [2,3,4]. The color change occurs because of the different electronic structures of the species involved in the reversible redox reaction. In the gel, the polymer or even the monomers before the polymerization assist the ions separation that occurs during the redox process. Different kinds of polymeric gel electrolytes have been deeply studied and characterized [3,4].

All solid electrochromic devices have a typical structure: two transparent substrates covered by electronic conductor layers (i.e., indium tin oxide, ITO), an electrochromic active material (working electrode), an ion conductive layer, and an ion storage layer (counter electrode), which can also be made using a complementary electrochromic material.

The electrochromic devices can be classified into three large families: inorganic, organic, and polymeric. The inorganic electrochromic materials, such as transition metal oxides (WO_3_ or V_2_O_5_) [5], are usually deposited on the two electrodes in the electrochromic device as a redox couple: i.e., while WO_3_ is reducing, the V_2_O_5_ oxidation occurs. Therefore, when the WO_3_ is in its reduced form, the device becomes blue [6].

Instead, the use of polymeric electrochromic materials, such as polyaniline (PANI) or polypyrrole (PPy) is very promising, for example, in the field of supercapacitors [7] or to improve the performance of the electrochromic devices in combination with metal oxides [8].

Anyway, today, organic compounds are the most used materials to prepare electrochromic devices; in particular, the 1,1′-disubstituted-4.4′-bipyridinum salts bipyridinium systems, also known as Viologens, are of great interest nowadays [9]. They can be defined as 1,1′-disubstituted-4.4′-bipyridinum salts and exist in dicationic form and can be reduced two times: the most stable reduced form is the radical one [9]. Ethyl viologen diperchlorate has been used to make electrochromic devices studied in the present work [10,11]. As stated above, the viologen can be involved in two reduction reactions: the first one leads to the formation of a radical species, while the second one can entirely reduce the viologen leading to a neutral species. It is well-known that the radical species of viologen is very stable and it shows a very intense blue coloration because of the radical electron delocalization of the π-system of the bipyridyl rings, while the more unstable neutral species has lower intensity coloration [12]. Viologen-based electrochromic devices could be very interesting to obtain flexible electrochromic devices.

Cyclic voltammetry is the main technique used to characterize the electrochemical properties of electrochromic devices [13,14]. In order to study the transmittance and the absorbance of the materials, the principal technique used has been UV-vis-NIR spectroscopy [15,16]. Raman spectroscopy has been used to characterize the materials used in the samples because it is well known that it is a powerful tool for molecular analysis and structural phases of many materials such as: graphene [17,18], polymers [19], thin films [20,21], cultural heritage [22], bio-systems [23] and so on [24,25,26].

The aim of this work is to investigate the performances of the electrochromic devices made with ethyl viologen diperchlorate, 1,1′-diethyl ferrocene (as a redox couple) and bisphenol A, at different concentrations of the electrochromic gel mixture and as a function of the polymerization of bisphenol A glycerolate (1 glycerol/phenol) diacrylate.

## 2. Results and Discussion

### 2.1. Cyclic Voltammetry Measurements

Cyclic voltammetry measurements have been performed to study the electrochemical properties of two different series of electrochromic devices based on two series of electrochromic gel polymer mixture: the first series is based on three percentages of the electrochromic gel mixture set at 55%, 60% and 65% and indicated as EM_55_, EM_60_ and EM_65_, containing the not-polymerized bisphenol A. The second set of samples was made using the same electrochromic gel mixture at the stated percentage concentrations, but the bisphenol A was polymerized.

The cyclic voltammograms (CVs) were collected on the devices with a scanning speed of 50 mV/s in the voltage range between −1.5 V and 1.5 V. The estimation of the working voltages of each device, the identification of the redox peaks and the study of the effect of polymerization of the bisphenol A on the electrochemical performances of the device were obtained.

**Figure 1:**
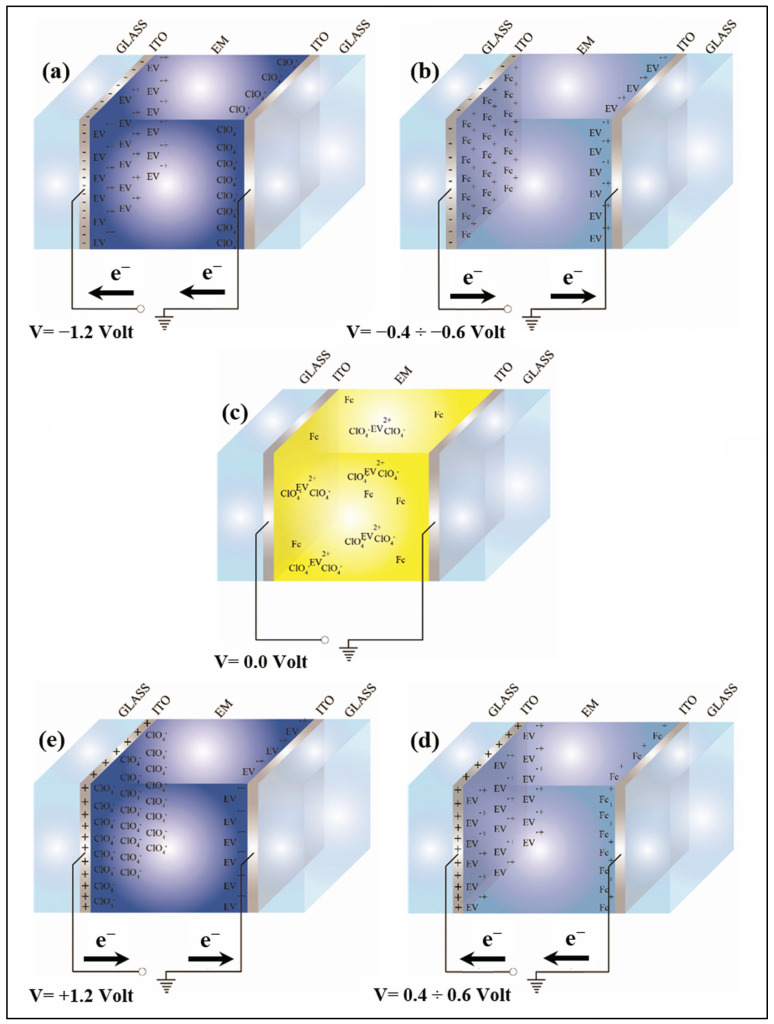
Schematic representation of electrochromic device at different applied voltages: −1.2 V (**a**), between −0.4 and −0.6 V (**b**), 0 V (**c**), between 0.4 V and 0.6 V (**d**) and 1.2 V (**e**) (Fc indicates 1,1′-diethyl ferrocene, EV is ethyl viologen).

Figure 1 represents an electrochromic device when different voltages are applied: 1.2 V, between −0.4 V and −0.6 V, 0 V, between 0.4 V and 0.6 V and 1.2 V. The species involved in the redox process between the two electrodes are also indicated. 

**Scheme 1:**
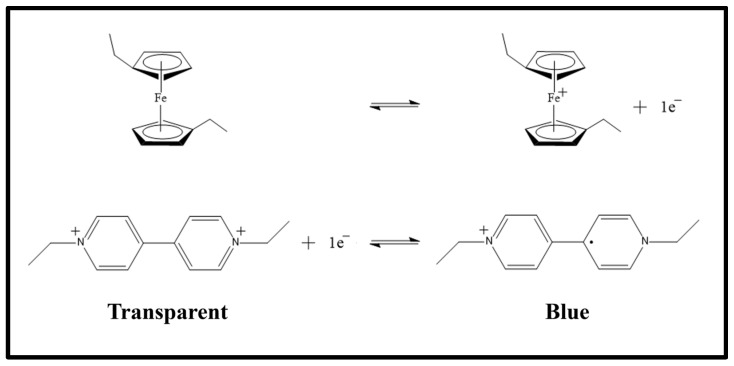
Schematic view of the redox reaction that occurs during the electrochromic process. In this scheme the two semi-reactions are shown: at the top the oxidation of the 1,1′-diethyl ferrocene and the reduction of the ethyl viologen diperchlorate. When the species are in the state shown on the left of the reactions, the electrochromic device is transparent; otherwise, when they are in the state on the right of the reactions, the devices becomes blue.

To better understand the schemes of Figure 1, it may be useful to look at Scheme 1, where the electrochromic reaction is drawn. In Scheme 1, the two semi-reactions are shown. In particular, at the top of the scheme, the oxidation of 1,1′-diethyl ferrocene is shown, which frees an electron that is used by the ethyl viologen diperchlorate in its dicationic form during the reduction reaction, at the bottom, which leads to the ethyl viologen diperchlorate radicalic monocationic form.

Figure 1a and Figure 1b show the same electrochromic conditions seen in Figure 1d and Figure 1e, respectively. The only difference is a change in the polarizability of the applied voltage: the electrode that before was positive becomes negative and vice versa. The charged species present in the gel move from one electrode to the other but no one reaction occurs. In Figure 1c the electrochromic species are dissolved in the gel and this situation is represented on the left side of Scheme 1, where the electrochromic device is transparent. When a voltage is applied to the device, then, depending on the sign of the applied voltage, one of the situations at the top or at the bottom of Figure 1 occurs, and the reactions are shifted to the right part of the scheme making the device blue.

When the voltage is removed, then, the device returns, spontaneously, to the transparent state (left side of the scheme).

**Figure 2:**
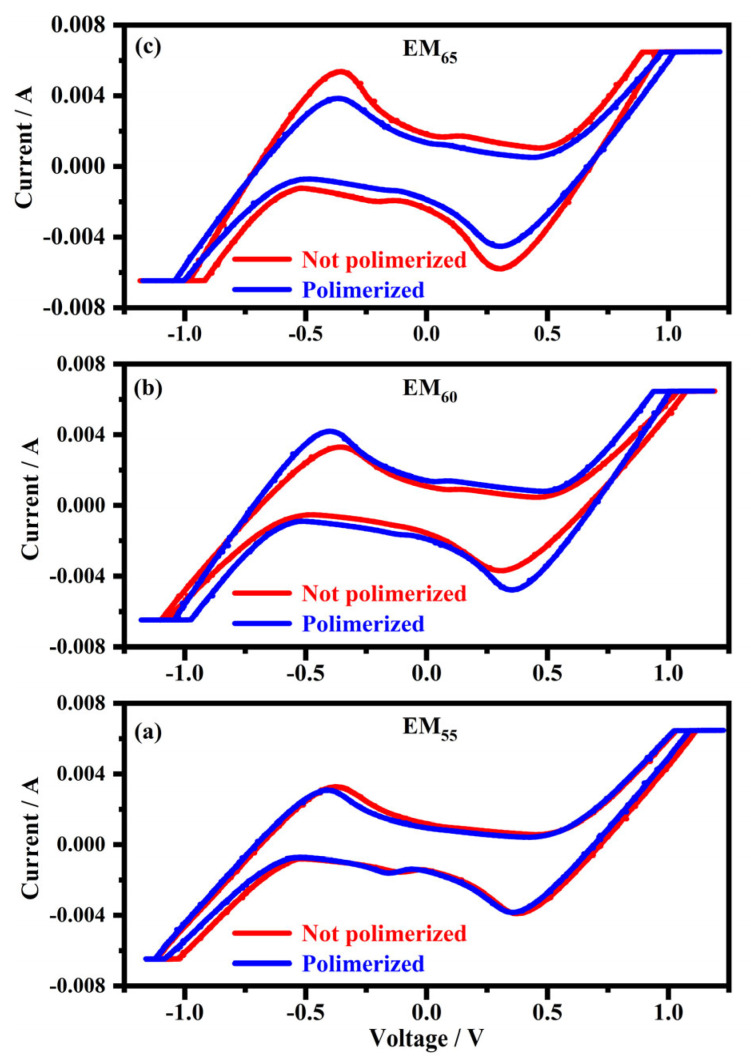
Cyclic voltammograms collected on the samples filled with the electrochromic gel mixtures at the weight ratios of 55% (**a**), 60% (**b**) and 65% (**c**), indicated as EM_55_, EM_60_ and EM_65_ with the bisphenol A not-polymerized (red line) and polymerized (blue line).

Figure 2 shows the cyclic voltammograms collected on the devices filled with the electrochromic gel mixtures in concentrations of 55%, 60% and 65%, labelled as EM_55_, EM_60_ and EM_65_, containing the bisphenol A polymerized and not-polymerized.

As exposed in Figure 2, cyclic voltammograms show the current maximum value, expressed at 6.5 × 10^−3^ A (in absolute value), inside the devices, while the switching potentials are comprised between 1.1 V and 1.2 V (in absolute value) to obtain the full coloration of the devices. The cyclic voltammograms recorded for all the mentioned electrochromic devices seem to be symmetric.

A first noticeable redox peak occurs at 0.4 V (in absolute value), related to 1,1′-diethyl ferrocene oxidation [12] and to the viologen reduction, while a peak comprised between 1.1 V and 1.2 V can be related to the viologen compound charge separation. It was reported that at specific potentials, the ionic bond between perchlorate ions and viologen (in its oxidized form) could be broken, causing an accumulation of charges on the electrodes and reducing the viologen [27]. Since ferrocene-based compounds are blue in the oxidized state and a radical form of a viologen compound with short alkyl substituents (such as ethyls) promotes a dark blue form [28,29], the blue color of the devices can be due to both the reactions: the oxidation of 1,1′-diethyl ferrocene and the reduction of the viologen species. Although the coloration process starts at voltages of about 0.4 V and becomes more intense at voltages between 1.1 V and 1.2 V, it does not end with the formation of radical species of viologen compound but with the formation of its neutral species. Nevertheless, the literature states that the double reduction reactions of viologens are irreversible [30]. At the same time, the system described here shows that reversibility and a dark blue, typical of a single reduced viologen, occurs. A lower intense peak can be noted at 0.2 volt potentials (in absolute value); a redox potential of 0.16 V was reported for reactions involving methyl viologen diperchlorate dimers [31].

As it can be seen in Figure 2, the switching potentials seem to become lower, passing from 55% to 65%. On the contrary, the current intensities shown by the different devices become higher, and the composition in the electrochromic solution passes from 55% to 65%. The effect of the polymerization is negligible in the sample with EM_55_. In the case of EM_60_, the not-polymerized samples show switching potentials higher than the polymerized samples, while in the case of EM_65_, the not-polymerized samples show a higher current than the polymerized ones.

### 2.2. UV-Vis-NIR Spectroscopy

Dynamic transmittance measurements were performed using UV-Vis-NIR spectroscopy to study the optical properties of the electrochromic devices filled with EM_55_, EM_60_ and EM_65_ with the polymerized and not-polymerized bisphenol A in the OFF/ON states. The transmittance curves were collected from 100 nm to 1310 nm, when the system was under different constant applied voltage, starting from 0.1 V to 1.2 V and increasing in steps of 0.1 V.

**Figure 3:**
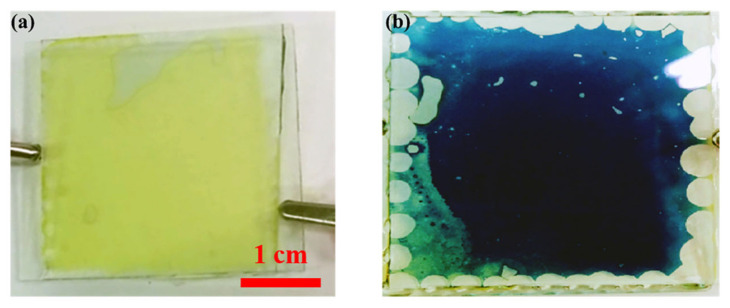
The electrochromic device in the OFF state (**a**) and ON state (**b**).

Figure 3 shows photographs of the electrochromic devices in their OFF state (bleached state) and ON state (blue-colored state) under the application of an external voltage.

In the presented electrochromic device, the transparent signs at the edges are due to the epoxy glue used to seal the device in the sandwich configuration. We are developing an automatic glue dispenser that controls the quantity of glue used at each point to avoid this effect. The glue will be placed on the inner face of one electrode, and the other electrode will be placed on top using automatic mechanical arms, to avoid any accidental signs of the glue.

It is possible to see in Figure 3 the presence of some bubbles at the edge of the device, which are due to the glue used to attach the electrodes of the device. It is planned to develop an automatic glue dispenser that will control the amount of the glue used during each point in order to avoid such an effect.

**Figure 4:**
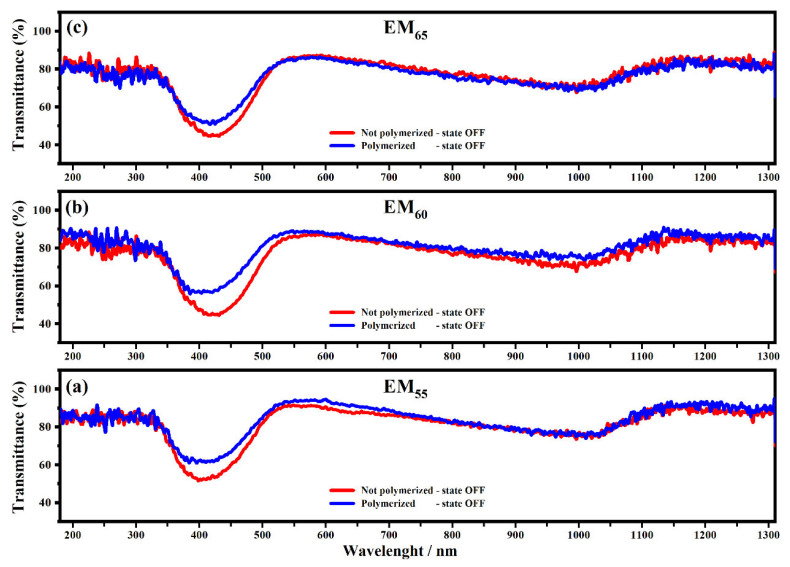
Transmittance spectra collected on the devices filled with the electrochromic gel mixtures: EM_55_ (**a**), EM_60_ (**b**) and EM_65_ (**c**), not-polymerized (red line) and polymerized (blue line), in the state OFF and in the range from 100 nm to 1310 nm at 0 V.

**Figure 5:**
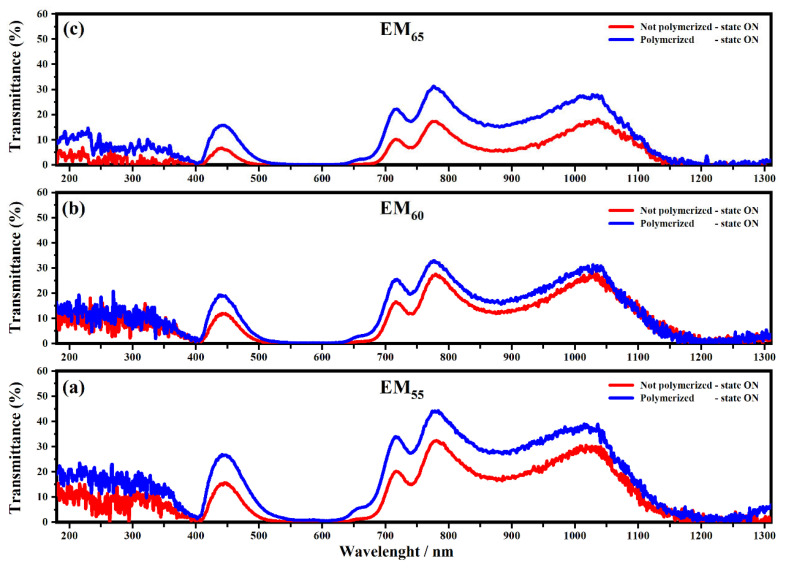
Transmittance spectra collected on the devices filled with the electrochromic gel mixtures: EM_55_ (**a**), EM_60_ (**b**) and EM_65_ (**c**), not-polymerized (red line) and polymerized (blue line), in the state ON and in the range from 100 nm to 1310 nm at 1.2 V.

Figure 4 and Figure 5 show the transmittance spectra collected on the devices filled with EM_55_, EM_60_ and EM_65_ in the OFF state (0 V) and in the ON state (1.2 V).

All spectra showed interesting variations of the transmittances in both OFF/ON states. As shown in Figure 4, the electrochromic devices are characterized by the first appreciable decrease of the transmittance, which falls at about 400 nm, and it is due to the presence of ethyl viologen radicals [29]. For the devices based on the not-polymerized EM_55_, the transmittance in the OFF state is equal to 50%, while in the case of the polymerized EM_55_, the transmittance is equal to 60%. For the devices based on the not-polymerized EM_60_, in the OFF state, the transmittance is equal to 45%, while in the case of the polymerized EM_60_, the transmittance is equal to 60%. The electrochromic devices containing the not-polymerized EM_65_ exhibit a transmittance of 50%, while the devices prepared with the polymerized EM_65_ exhibit a transmittance of 60%. No appreciable variations of transmittance were found at the wavelengths of 550 nm, 880 nm and 1200 nm. In Figure 5, at the wavelengths of 550 nm, 880 nm and 1200 nm, a decrease of the transmittances can be observed for all kinds of electrochromic devices, and this behavior is justified by the formation of the radical species of the ethyl viologen, after which the redox process occurred. In the ON state, all the devices made using the not-polymerized and polymerized gel mixtures EM_55_, EM_60_ and EM_65_ are characterized by values of transmittances at about 0%. At 880 nm, the transmittance of devices with the not-polymerized EM_55_ is about 20%, while for the polymerized EM_55_, it is equal to 30%. At 880 nm, the transmittance of the devices containing the not-polymerized EM_60_ is about 15%, while the devices with the polymerized EM_60_ show a decrease of the transmittance of 20%. The devices composed by the not-polymerized EM_65_ show a value of transmittance of 10% in the ON state, and with the polymerized EM_65_, the transmittance is equal to 20%, compared to the OFF state. Since there is no evidence of the oxidized ferrocene compound’s characteristic green coloration (when they are in a ferrocene-based solution) [31], one can conclude that 1,1′-diethyl ferrocene oxidation and ethyl viologen diperchlorate reduction occur at the same potential.

**Figure 6:**
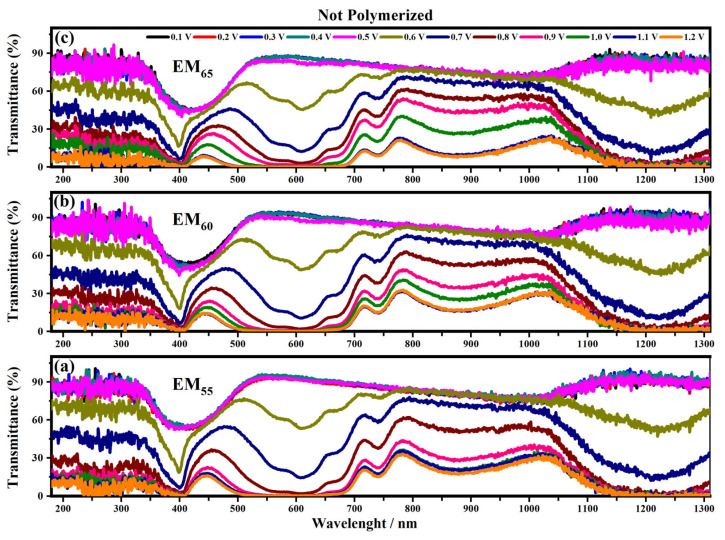
Transmittance spectra collected on the devices prepared with the not-polymerized electrochromic gel mixtures, EM_55_ (**a**), EM_60_ (**b**) and EM_65_ (**c**), at different applied voltages comprised between 0.1 V and 1.2 V.

**Figure 7:**
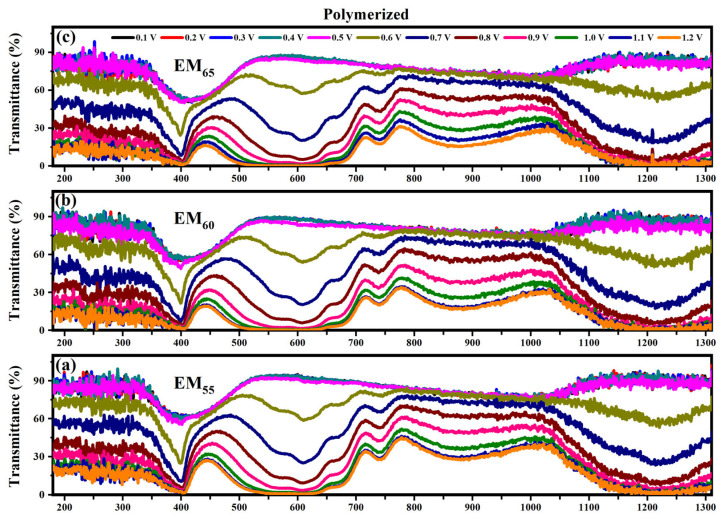
Transmittance spectra collected on the devices prepared with the polymerized electrochromic gel mixtures, EM_55_ (**a**), EM_60_ (**b**) and EM_65_ (**c**), at different applied voltages comprised between 0.1 V and 1.2 V.

Figure 6 and Figure 7 show the transmittance spectra of the electrochromic devices made using EM_55_, EM_60_ and EM_65_ (containing the not-polymerized and polymerized bisphenol A gels) when each system was in its ON state under an applied voltage from 0.1 V to 1.2 V.

A noticeable change of transmittance in the visible and infrared regions can be seen for voltages between 0.5 V and 0.6 V. This behavior can be associated with the starting of the redox process: i.e., the oxidation of the 1,1′-diethyl ferrocene and the reduction of the ethyl viologen diperchlorate. In particular, for the devices containing the not-polymerized EM_55_ gel the first appreciable transmittance variation is found under the application of 0.6 V. The transmittance of these kind of devices is about 45% until 0.5 V, then the transmittance varies from 15% (at 0.6 V) to 0% (at 1.2 V) at 400 nm. At 550 nm, the not-polymerized EM_55_ devices show 90% of transmittance until 0.5 V, then a decrease of up to 60% is found at 0.6 V and a value of 0% at 1.2 V. At 880 nm, the not-polymerized EM_55_ devices show 75% of transmittance until 0.5 V, then a decrease up to 15% is found at 1.2 V. At 1200 nm, the transmittance values for the not-polymerized EM_55_ devices vary from 90% (until 0.5 V) to 60% (at 0.6 V) and to 0% (1.2 V).

For the devices based on the not-polymerized EM_60_ gel devices at 400 nm, the transmittance changes from 45% (until 0.5 V) to 15% (at 0.6 V) and then to 0% at 1.2 V. The same kind of devices at 550 nm, exhibit a variation of the transmittances from 90% (until 0.5 V) to 45% (at 0.6 V) and to 0% (at 1.2 V), while at 880 nm, the transmittance changes from 80% at 0.6 V to 15% at 1.2 V. At 1200 nm, the transmittance is stable at 90% until 0.5 V, and then it decreases to 45% at 0.6 V and to 0% at 1.2 V. The systems containing the not-polymerized EM_65_ gel at 400 nm exhibit a variation of the transmittances from 60% (until 0.5 V) to 20% at 0.6 V and to 0% at 1.2 V. At 550 nm, these systems are characterized by 90% of transmittance up to 0.5 V, and then a decrease up to 50% is found at 0.6 V. At 880 nm, the transmittance of these devices varies from 75% (until 0.6 V) to 15% at 1.2 V, and at 1200 nm, the devices show 90% of transmittance until 0.5 V, and then a decrease up to 45% under the application of 0.6 V and about 0% at 1.2 V were observed.

The electrochromic devices based on the polymerized EM_55_, EM_60_ and EM_65_ gel mixtures show a variation of the transmittance at 400 nm from 60% (until 0.5 V) to 30% (at 0.6 V) and to 0% at 1.2 V. At 550 nm, the transmittance changes from 90% (until 0.5 V) to 60% (at 0.6 V) and to 0% at 1.2 V. At 880 nm, for EM_55_, the transmittance changes from 90% (until 0.5 V) to 15% (at 1.2 V), and for EM_60_ and EM_65_, the transmittances vary from 90% (until 0.5 V) to 15% (1.2 V). At 1200 nm, the transmittances vary from 90% up to 0.5 V, and then after the application of 0.6 V, a decrease at around 60% is reached, and at 1.2 V, the devices show transmittances of about 0%.

This agrees with cyclic voltammetry measurements, which confirmed that the maximum current is achieved at 1.2 V, when perchlorate ions and viologen dications separate and diffuse to the electrodes.

Focusing on the highest value of voltage (1.2 V), the time required for the switching ON of the devices and, consequently, the evaluation of the memory effect of the devices have been studied.

**Figure 8:**
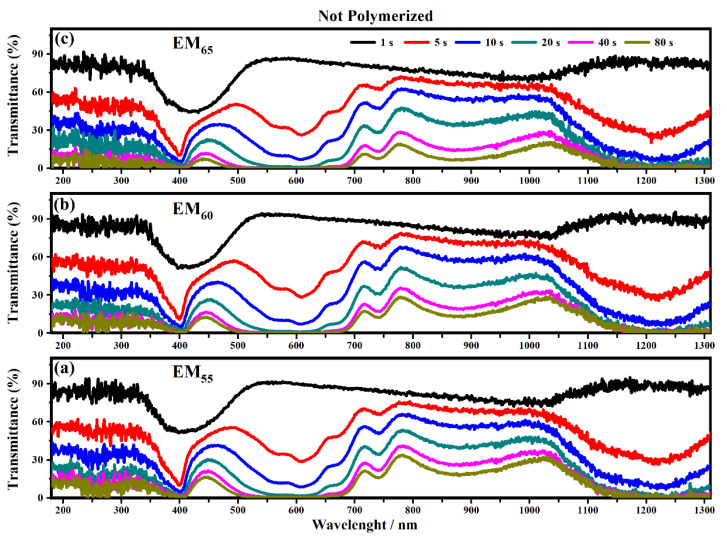
Transmittance spectra collected on the electrochromic devices filled with the not polymerize electrochromic gel mixtures, EM_55_ (**a**), EM_60_ (**b**) and EM_65_ (**c**), under the application of a voltage of 1.2 V to switch ON the devices.

**Figure 9:**
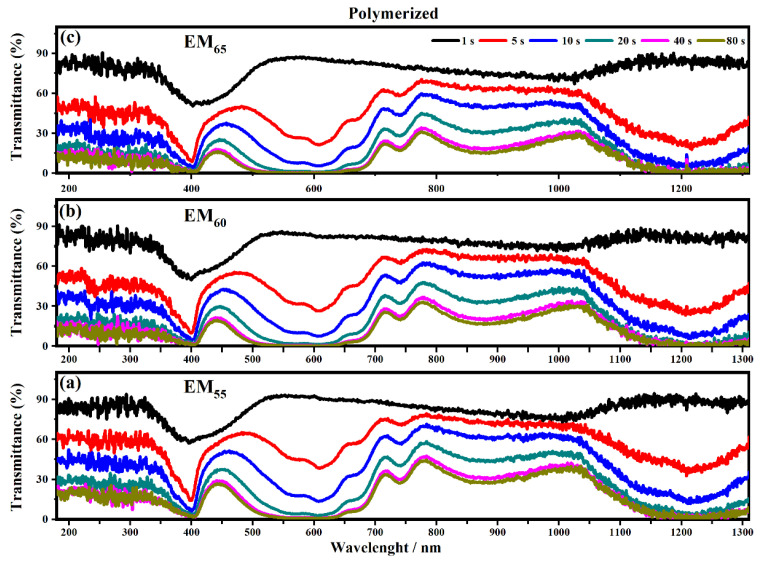
Transmittance spectra collected on the electrochromic devices filled with the polymerized electrochromic gel mixtures, EM_55_ (**a**), EM_60_ (**b**) and EM_65_ (**c**), under the application of a voltage of 1.2 V to switch ON the devices.

Figure 8 and Figure 9 show the transmittance spectra of the electrochromic devices containing the not-polymerized and polymerized EM_55_, EM_60_ and EM_65_ gels when the devices are switched from the OFF to the ON state.

As shown in Figure 8 all the devices based on the not-polymerized electrochromic gel mixtures (EM_55_, EM_60_ and EM_65_) need 5 s to switch from the OFF to the ON state. The same time of 5 s was found for electrochromic devices containing the polymerized electrochromic gel mixtures EM_55_, EM_60_, and EM_65_ to obtain the switching between the OFF/ON states under the maximum values of applied potential (1.2 V). In both series of electrochromic devices, the highest decreases of transmittance were found after 80 s.

**Figure 10:**
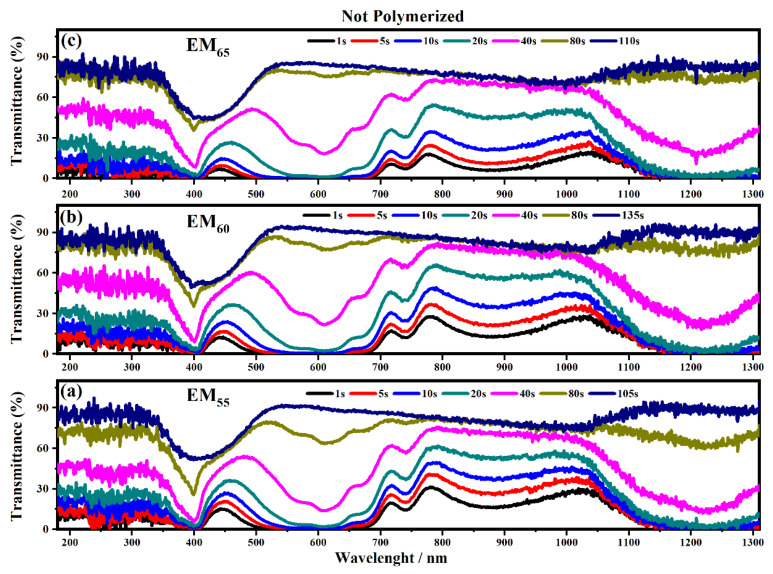
Transmittance spectra collected on the electrochromic devices filled with EM_55_ (**a**), EM_60_ (**b**) and EM_65_ (**c**) not-polymerized gels under a voltage of 1.2 V to switch OFF the devices.

**Figure 11:**
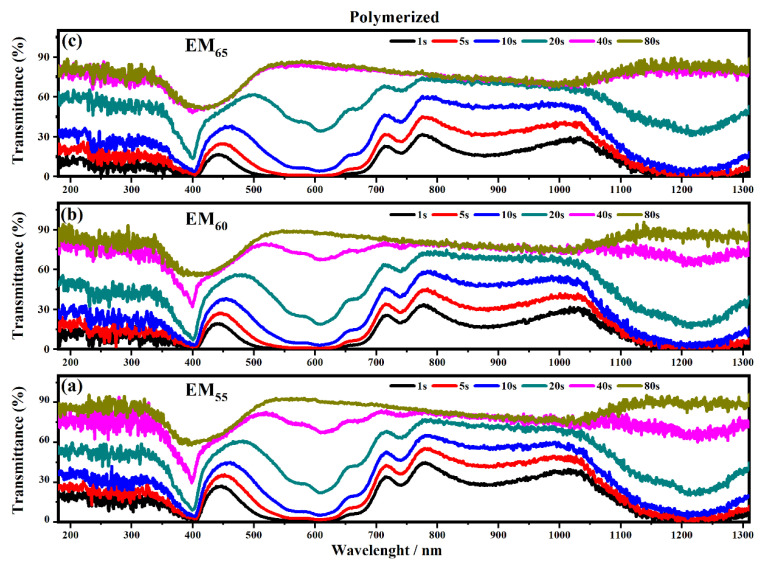
Transmittance spectra collected on the electrochromic devices filled with EM_55_ (**a**), EM_60_ (**b**) and EM_65_ (**c**) polymerized gels under a voltage of 1.2 V to switch OFF the devices.

Figure 10 and Figure 11 show the transmittance spectra collected on the electrochromic devices, based on the not-polymerized and polymerized electrochromic gel mixtures (EM_55_, EM_60_ and EM_65_) when the devices are switching from the ON to the OFF state.

The polymerization process of the bisphenol A seems to improve the switching of the devices between the ON/OFF states. As shown in Figure 10, the electrochromic devices containing the not-polymerized bisphenol A took 40 s in order to switch from the ON to the OFF state, and at the end, the devices are completely switched OFF after a time of between 80 and 135 s.

In particular, the device with EM_55_ needs 105 s to switch OFF, while the devices based on EM_60_ and EM_65_ need a time of 135 s and 110 s, respectively. After the polymerization process of bisphenol A, all the devices need 80 s to switch OFF.

**Figure 12:**
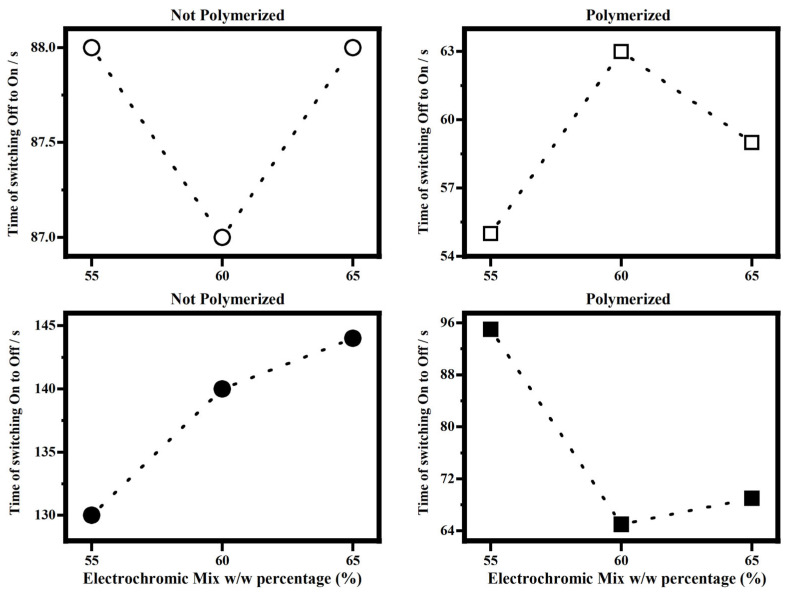
Time of switching OFF/ON and ON/OFF vs. percentage w/w mix of electrochromic/bisphenol A, EM_55_, EM_60_ and EM_65_ gels of the electrochromic devices not-polymerized (on the left) and polymerized (on the right).

Figure 12 shows the times of the switching starting by the state OFF to the state ON and starting to state On to the state OFF, for the electrochromic devices filled with the polymerized and not-polymerized EM_55_, EM_60_ and EM_65_ gels to compare the effect of the polymerization on such response times.

As it can be seen in Figure 12, once a voltage of 1.2 V is applied, the times for the switching OFF/ON of the electrochromic devices filled with not-polymerized EM_55_, EM_60_ and EM_65_ gels are about 88 s, while for the polymerized samples, the switching time falls in the range between 55 s and 63 s. As stated above, the memory times in not-polymerized devices are higher than those in polymerized. In fact, for the not-polymerized devices, the memory times fall in the range between 130 s and 145 s, while for the polymerized ones, they fall in the range between 64 s and 96 s. To reach full coloration, electrochromic devices filled with EM_55_, EM_60,_ and EM_65_ polymerized gels are faster than the respective not-polymerized devices (Figure 12). Since the effect of the polymerization is to decrease the resistance to the ions diffusion inside the devices, it affects the memory times, which are lower for polymerized systems than for not-polymerized.

In both the systems, polymerized and not-polymerized, the spectra collected in the devices with the highest concentration of the electrochromic materials show a lower transmittance, at each wavelength, in both the states OFF and ON. Of course, it can be assigned to the number of the color centers that increase, as well as the concentration of the electrochromic materials.

The coloration efficiency (CE) at a given wavelength is an important parameter to establish how good a device is (Equation (1)):

(1) $$\;\;CE=\frac{∆A}{Q}$$

In the previous formula, ΔA is the absorbance difference between ON and OFF states, and Q is the number of charges injected per unit area ([C]/[cm]^2^).

The values of CE at 580 nm and at 1200 nm for the devices filled with the polymerized and not-polymerized electrochromic gel mixtures (EM_55_, EM_60,_ and EM_65_) are reported in Table 1.

**Figure 13:**
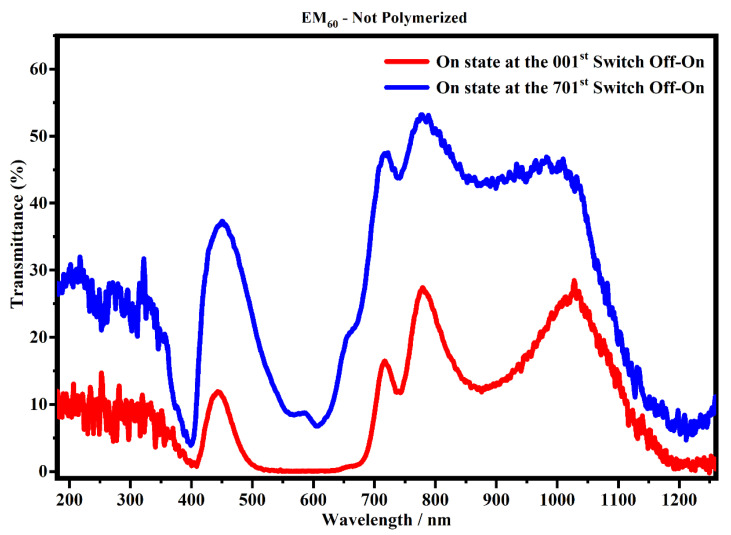
Transmittance spectra, in the state ON, of the devices filled with the EM_60_ not-polymerized gel at the 1st (red line) and 701st cycle (blue line).

**Figure 14:**
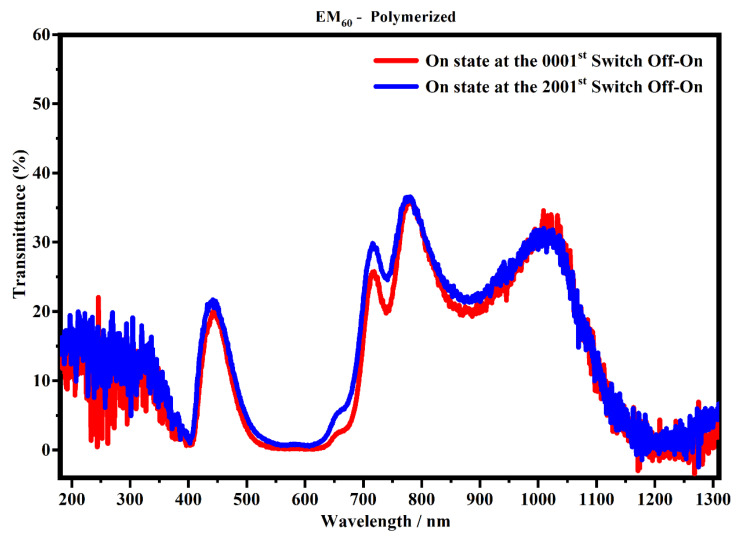
Transmittance spectra, in the state ON, of the devices filled with the EM_60_ polymerized gel at the 1st (red line) and 2001st cycle (blue line).

Focusing on the devices made of the electrochromic gel mixture at a concentration of 60% (EM60), containing the not-polymerized and the polymerized bisphenol A, Figure 13 and Figure 14 show the transmittances recorded after the first cycle and after 700 cycles (one cycle correspond to the two processes: switch OFF/ON and switch ON/OFF).

Figure 13 shows the transmittance spectra collected on the devices filled with EM_60,_ not-polymerized gel after the first cycle and after 700 cycles (switch OFF/ON and switch ON/OFF).

Figure 13 shows that the spectra collected after 700 cycles have a lower transmitance than the spectra collected after one cycle, and in particular, ΔT% at 400 nm, 600 nm and 1205 nm is 3.3%, 6.6% and 6.3%, respectively. This means that the sample could suffer a possible degradation of the gel mixture subjected to an external applied voltage. On the contrary, the system which contains the polymerized EM_60_ is still stable up to 2000 cycles, as shown in Figure 14. In particular, it can be noticed that the polymerized devices do not exhibit appreciable variation of transmittance in the considered range. The polymerization process and the combination of the gel polymer mixture play a key role in enhancing the life of electrochromic devices.

The novelty of these devices is using a polymeric gel mixture made using a viologen and ferrocene couple, and bisphenol A can assist in the involved redox process. An essential feature of the electrochromic device is the high transparency and the possibility of using this unique feature to fabricate smart windows compared to other devices studied in the literature [32,33,34]. In addition, the fabricated ECDs revealed interesting optical properties, as confirmed via UV-vis spectroscopy and independent color-switching reversible coloration and bleaching cycles, making them useful for coloration efficiency and promoting energy-saving buildings.

### 2.3. Raman Spectroscopy

Raman spectroscopy was performed on the electrochromic devices filled with the polymerized and not-polymerized electrochromic gel mixtures (EM_55_, EM_60_ and EM_65_).

**Figure 15:**
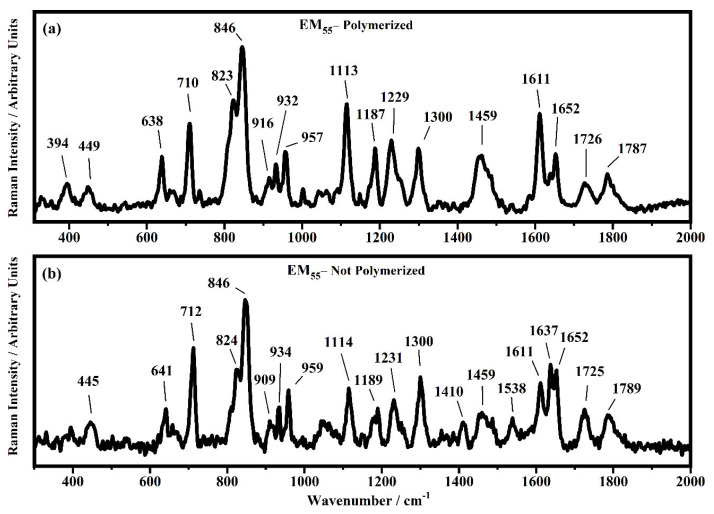
Raman spectra collected in the range between 310 and 2000 cm^−1^ on the samples EM_55_ polymerized (**a**) and not-polymerized (**b**).

**Figure 16:**
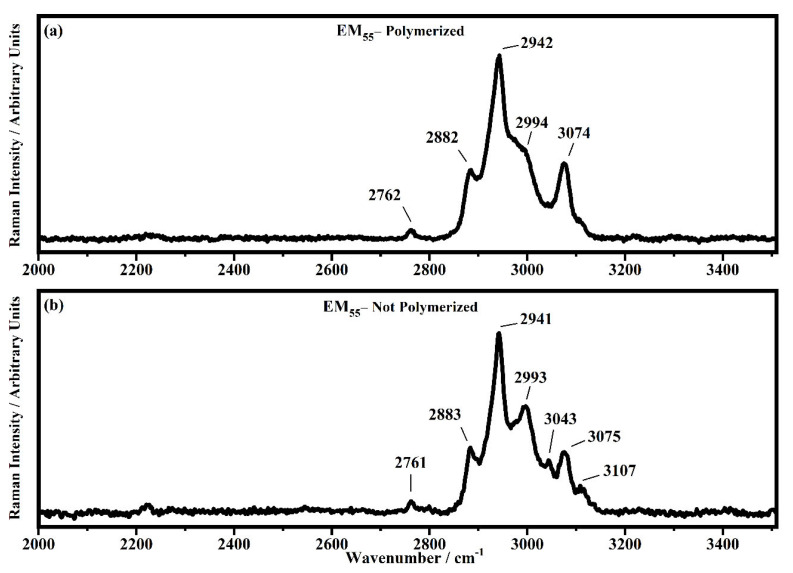
Raman spectra collected in the range between 2000 and 3510 cm^−1^ on the samples EM_55_ polymerized (**a**) and not-polymerized (**b**).

**Figure 17:**
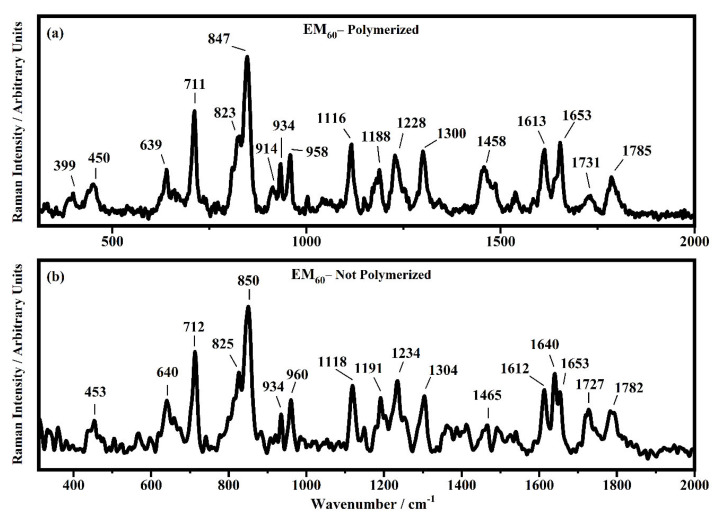
Raman spectra collected in the range between 310 and 2000 cm^−1^ on the samples EM_60_ polymerized (**a**) and not-polymerized (**b**).

**Figure 18:**
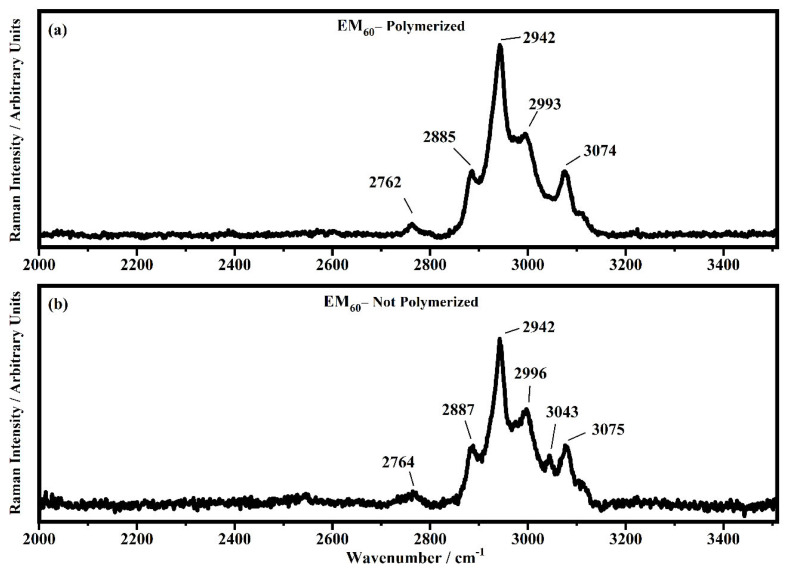
Raman spectra collected in the range between 2000 and 3510 cm^−1^ on the samples EM_60_ polymerized (**a**) and not-polymerized (**b**).

**Figure 19:**
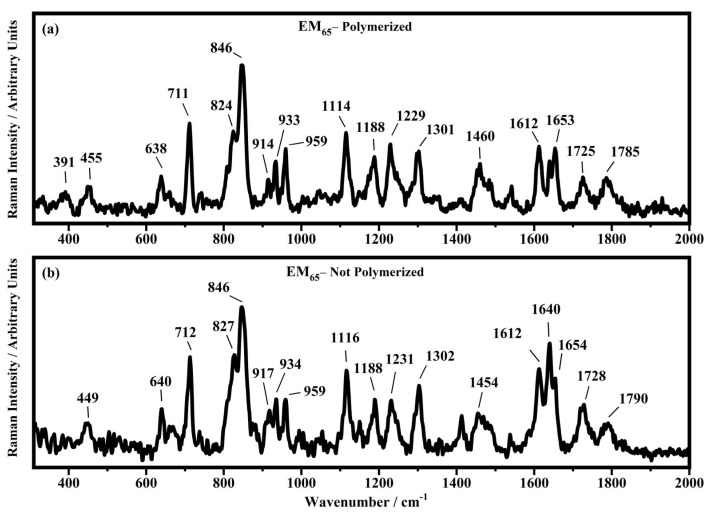
Raman spectra collected in the range between 300 and 2000 cm^−1^ on the samples EM_65_ polymerized (**a**) and not-polymerized (**b**).

**Figure 20:**
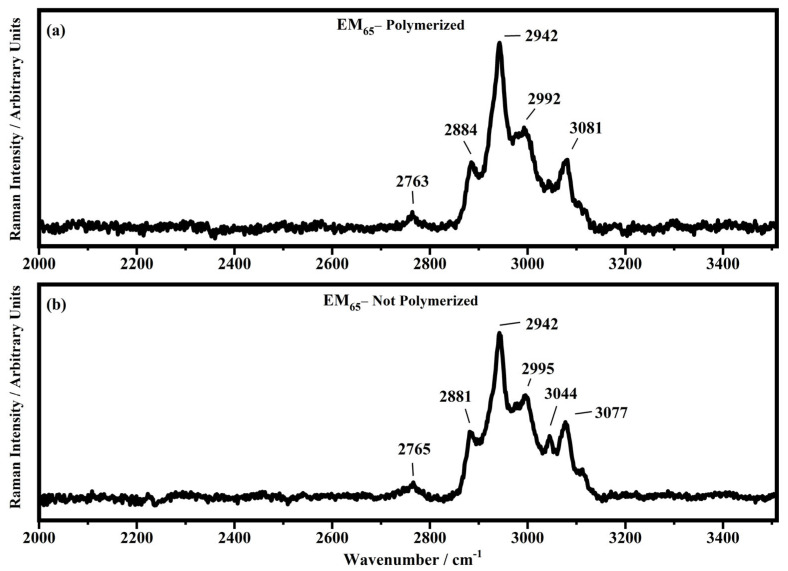
Raman spectra collected in the range between 2000 and 3510 cm^−1^ on the samples EM_65_ polymerized (**a**) and not-polymerized (**b**).

Raman spectra are shown in two ranges: from 310 cm^–1^ to 2000 cm^−1^ and from 2000 cm^−1^ to 3510 cm^−1^. In Figure 15, Figure 16, Figure 17, Figure 18, Figure 19 and Figure 20, the Raman spectra collected on the electrochromic devices are shown.

All the Raman spectra show similar peaks. Very low Raman peaks below 455 cm^−1^ can be assigned to 1,1′-diethyl ferrocene ring modes. The bands comprised between 822 cm^−1^ and 826 cm^−1^ can be assigned to the ring breathing mode of the ethyl viologen diperchlorate. The triplet centered around 935 cm^−1^ is related to the Cl–O stretching in the perchlorate ions when they are free, which interact with propylene carbonate or with each other [27,35]. Several signals in the spectra can be assigned to propylene carbonate [36]: the band at 455 cm^−1^ can be assigned to the propylene carbonate ring deformation, while the strong band at 712 cm^−1^ can be assigned to its symmetric ring deformation. The strong band at 849 cm^−1^ can be assigned to the C–H methyl group and to O–C–C (not comprising a carbon bond to the methyl group) bending of propylene carbonate. The band at 1119 cm^−1^ is related to the C–H methyl group and to the C–H stretching of carbon of the methyl group of propylene carbonate. A very low signal can be identified at about 1147 cm^−1^, corresponding to the stretching of the C–C bond and to the bending of the O–C–O group of the propylene carbonate.

Some Raman bands comprised between 1336 cm^−1^ and 1391 cm^−1^ could be assigned to the bending of the C-H group of the propylene carbonate. Signals with very low intensity can be identified between 1336 cm^−1^ and 1391 cm^−1^, related to the C–H methyl group bending of propylene carbonate. The vibrational mode of 1460 cm^−1^ can be assigned to the umbrella mode of the C–H methyl group in the propylene carbonate. The Raman bands at 1726 cm^−1^ and 1787 cm^−1^ are ascribed to the stretching of the C=O groups: the first one is assigned to the acrylate carbonyl group and the second one to the carbonyl of the propylene carbonate.

In the region comprised between 2000 cm^−1^ and 3500 cm^−1^, a change in the Raman spectra can be noticed between the spectra collected on the polymerized and not-polymerized devices. The signal at about 3043 cm^−1^ in the spectra of the polymerized samples is very weak or totally absent compared to the spectra of not-polymerized devices. Such behavior is similar to the peak at 1640 cm^−1^ because both are related to the C=C stretching of the acrylate group of bisphenol A. The bisphenol A polymerization occurs at the terminal C=C double bond; studies of the intensity variation of the 1640 cm^−1^ bands during polymerization have already been reported [36].

Signals at ~2882 cm^−1^, ~2940 cm^−1^ and ~2990 cm^−1^ can be assigned to the various CH_2_ and CH_3_ stretching modes of species in the solution. The band at 3073 cm^−1^ can be assigned to the C-H stretching of aromatic rings. Since no signals can be observed in the 3200 cm^−1^–3500 cm^−1^ range, condensation between different polymeric chains can be excluded (i.e., no water can be identified in the spectra).

## 3. Conclusions

In this work, a study of electrochromic devices based on ethyl viologen diperchlorate and 1,1′-diethyl ferrocene as a redox couple in propylene carbonate has been outlined. Electrochromic devices were made using viologen-based electrochromic gel mixtures at concentrations of 55%, 60% and 65%, with the first set of samples containing not-polymerized bisphenol A and the second set polymerized bisphenol A.

The effects of the different concentration ratios and of the state of the bisphenol A (not-polymerized or polymerized) on the electrochromic performances of the devices have been shown.

Several characterizations, such as cyclic voltammetry measurements, UV-vis and Raman spectroscopies, clarified that the devices based on the concentration of 60 w/w% of electrochromic solution revealed engaging performances useful for the implementation of electrochromic devices for energy-saving applications.

Cyclic voltammetry measurements confirmed the reversible electrochemical behavior of the electrochromic devices based on the investigated electrochromic gel mixtures.

The redox peak at about 0.4 V was assigned to the oxidation peak of the 1,1′-diethyl ferrocene oxidation and to the reduction of the ethyl viologen. The potentials needed to switch on the devices seems to be lower and the composition in the electrochromic solution passes from 55% to 65%. In comparison, the current intensities present in the different devices become higher and the composition in the electrochromic solution passes from 55% to 65%. The effect of the polymerization is negligible in the sample with EM_55_. In the case of EM_60_, the not-polymerized samples show switching potentials higher than the polymerized samples, while in the case of EM_65_, the not-polymerized samples show a higher current with respect to the polymerized ones.

At 550 nm, 880 nm and 1200 nm, decreases of the transmittances can be observed for all electrochromic devices, and the formation of the radical species of the ethyl viologen justifies this behavior. Focusing on the devices EM_60_, after the polymerization of the bisphenol A, the transmittance changed from 90% to 60% at 0.6 V and reached 0% at 1.2 V, at 550 nm. At 880 nm, the transmittances varied from 90% (until 0.5 V) to 15% (1.2 V), and at 1200 nm, the devices showed a variation from 90% until 0.5 V to 60% at 0.6 V and, at the end, to about 0% at the maximum voltage. At 1.2 V, the devices with not-polymerized bisphenol A and based on the mixture at a concentration of 60% (EM_60_) took a time of 63 s to switch OFF/ON and 60 s to switch ON/OFF.

The polymerization of bisphenol A helped to shorten the switching times and lengthen the cycle life of the investigated electrochromic devices (more than 2000 cycles), making them suitable devices for fabrication of smart windows.

EM_60_ devices can be used for energy-saving applications in buildings, with a promising CE of 92.82 C/cm^2^ in the visible region and 80.38 C/cm^2^ in the near infrared region.

## 4. Materials and Methods

An electrochromic solution (ES) based on the redox couple 1,1′-diethyl ferrocene and ethyl viologen diperchlorate (EV) was prepared in an aprotic solvent, propylene carbonate (PC, 99%), to assist the ions separation inside the electrochromic device. A second solution made of bisphenol A glycerolate (1 glycerol/phenol) diacrylate and the photo-initiator Irgacure (dimethoxy-phenylacetophenon 99%) was prepared and added to the first solution to obtain three electrochromic gel mixtures (EM) at the weight ratios of 55%, 60% and 65% (EM_55_, EM_60_ and EM_65_). Another series of the electrochromic gel mixtures was prepared at the same weight ratios of 55%, 60% and 65% (EM_55_, EM_60_ and EM_65_), after which the polymerization process of the bisphenol A occurred.

The electrochromic devices were fabricated using two transparent electrodes of Indium Tin Oxide (ITO)-coated glass, separated by silica sphere spacers with a diameter of 88 μm. The electrochromic devices were sealed using an epoxy resin and were exposed to the UV lamp light (from Jelosil, Milan, Italy, HG 200 L model, optical power per unit area 0.043 W/cm^−2^) for 1 min and 30 s. Each electrochromic device was filled with the electrochromic gel mixtures via capillarity.

**Figure 21:**
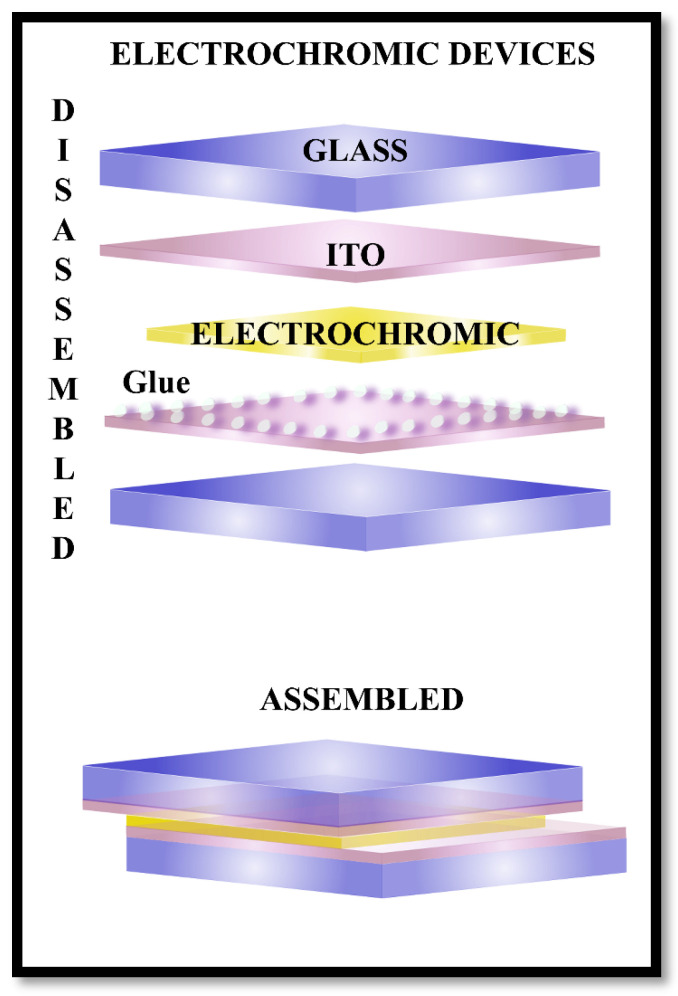
Electrochromic device disassembled (on the top) and assembled (on the bottom).

Figure 21 shows the main components of an electrochromic device and the final assembled device.

**Figure 22:**
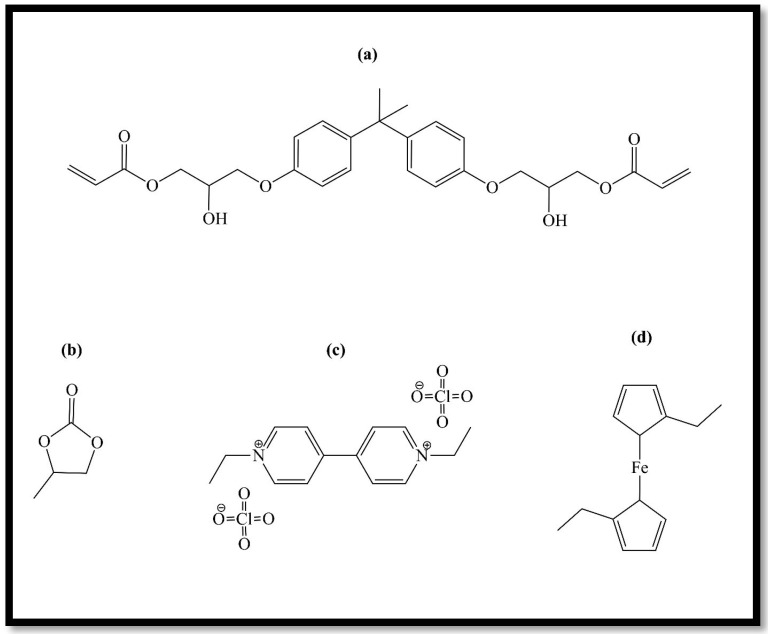
Electrochromic gel chemicals: (**a**) bisphenol A glycerolate (1 glycerol/phenol) diacrylate, (**b**) propylene carbonate; (**c**) ethyl viologen diperchlorate; (**d**) diethyl ferrocene.

All chemicals, shown in Figure 22, were purchased from Sigma−Aldrich Merk, Milan, Italy and used without any particular additional treatments, except for bisphenol A glycerolate (1 glycerol/phenol) diacrylate. Since this compound has too high a density even at room temperature, a heat treatment was necessary to manipulate it [3].

Transmittance measurements were performed using a spectrophotometer (from AvaSpec supplied by Avantes, Apeldoorn Netherlands), 2048-Avantes equipped with a Deuterium-Halogen light source (DH-2000 UV-Vis-Nir Lightsource Avantes) and Agilent E3632A DC Power Supply (0-15V, 7A), Agilent Technologies, Palo Alto, CA, USA

Cyclic voltammetry measurements were performed using a potentiostat/galvanostat (model 2059 from Amel Instrument s.r.l. Perigny, France) equipped with the 7800 interface in order to manage measurements using Junior Assist software 2059.

Raman spectroscopy measurements were performed using a micro-Raman-LABRAM (from Horiba-Jobin Yvon Srl, Piscataway, NJ, USA) equipped with an optical microscope in order to collect Raman spectra from small regions of the sample, such as 2–5 µm. MicroRaman-LABRAM is equipped with a HeNe 17 mW laser (633 nm), a NdYAG 50 mW laser (doubled, 532 nm) and a CCD detector (Peltier cooled, 1024 × 256). The optical microscope is equipped with 10× NA 0.25, 50× NA 0.7 and 100× NA 0.9 objectives.

## Figures and Tables

**Table 1:** Coloration efficiency at 580 nm and 1200 nm of the electrochromic devices filled with EM_55_, EM_60_ and EM_65_.

Sample	Not-Polymerized		Polymerized	
	CE [C]/[cm]^2^ (at 580 nm)	CE [C]/[cm]^2^ (at 1200 nm)	CE [C]/[cm]^2^ (at 580 nm)	CE [C]/[cm]^2^ (at 1200 nm)
EM_55_	101.03	100.93	27.12	24.07
EM_60_	93.61	65.03	92.82	80.38
EM_65_	42.83	71.96	54.76	56.90

## Data Availability

The data presented in this study are available upon request to the corresponding author.
